# Improved assessment of middle ear recurrent/residual cholesteatomas using temporal subtraction CT

**DOI:** 10.1007/s11604-021-01209-2

**Published:** 2021-10-24

**Authors:** Akira Baba, Satoshi Matsushima, Takeshi Fukuda, Hideomi Yamauchi, Hiroaki Fujioka, Jun Hasumi, Shohei Yoshimoto, Tomokazu Shoji, Sho Kurihara, Yutaka Yamamoto, Hiromi Kojima, Ryo Kurokawa, Mariko Kurokawa, Yoshiaki Ota, Hiroya Ojiri

**Affiliations:** 1 Department of Radiology, The Jikei University School of Medicine, 3-25-8 Nishi-Shimbashi, Minato-ku, Tokyo, 105-8461 Japan; 2 Division of Neuroradiology, Department of Radiology, University of Michigan, 1500 E. Medical Center Dr., Ann Arbor, MI 48109 USA; 3 Department of Otorhinolaryngology-Head and Neck Surgery, University of Miyazaki, 5200 Kihara, Kiyotake, Miyazaki, Miyazaki 889-1692 Japan; 4 Department of Otorhinolaryngology, The Jikei University School of Medicine, 3-25-8 Nishi-Shimbashi, Minato-ku, Tokyo, 105-8461 Japan

**Keywords:** Cholesteatoma, Middle ear, Recurrence, Computed tomography, Subtraction

## Abstract

**Purpose:**

The purpose of this study was to investigate the usefulness of temporal subtraction CT (TSCT) of temporal bone CT for the detection of postoperative recurrent/residual cholesteatoma of the middle ear.

**Methods:**

Thirty-two consecutive patients with surgically proven postoperative recurrent/residual cholesteatoma and 14 consecutive patients without recurrent/residual lesion matched the selection criteria and were retrospectively evaluated. TSCT imaging was generated with the use of serial postoperative CT. Two experienced radiologists and two residents evaluated the presence of bone erosive change by comparison serial CT studies, and CT and TSCT. The detection rate of bone erosive change, sensitivity and specificity of the recurrence/residual lesions, and reading time for each reader were evaluated.

**Results:**

TSCT + CT significantly improved the detection of bone erosive changes compared to CT-only evaluation (17.4–41.3% vs. 37.0–58.7%, *p* = 0.008–0.046). The mean sensitivity and specificity of TSCT + CT for experienced radiologists were 0.77 and 1.00, and 0.52 and 0.97 without TSCT. The mean sensitivity and specificity of TSCT + CT for residents were 0.64 and 1.00, and 0.41 and 1.00 without TSCT. Sensitivity showed an increase in all readers. The use of TSCT significantly reduced the reading time per case in all readers (*p* < 0.001).

**Conclusion:**

TSCT improves the depiction of newly occurring progressive bone erosive changes, and detection sensitivity and reading time in postoperative recurrence/residual cholesteatoma of middle ear.

## Introduction

Cholesteatoma is a benign collection of keratinized squamous epithelium of the middle ear. High-resolution CT (HRCT) is useful for the preoperative assessment of the extension and bone erosion of cholesteatoma, and MRI, especially non-echo-planar DWI (non-EPI DWI), has been reported to be useful in the preoperative diagnosis of cholesteatoma and for the evaluation of recurrence and residual lesions [1, 2]. In DWI, recurrent or residual cholesteatoma lesions are detected as markedly high signals and are relatively easy to diagnose. Postoperative CT of middle ear cholesteatoma is performed for the detection of recurrent/residual cholesteatoma, in addition to a detailed evaluation of the complex anatomy of the ossicles and their reconstruction, facial nerve canal, and inner ear. However, CT is shown to be unreliable in differentiating between recurrent/residual lesions, inflammation, fibrosis, granulation tissue, and cholesterol granuloma [3, 4]. In clinical practice, the diagnosis of recurrent/residual lesions cannot be determined without detecting progressive bone erosive changes as well as increased soft tissue density. In addition, interpretation of the temporal bone CT, with its complex anatomy and microstructures, is labor-intensive and depends on the experience of the reader. Recently, several studies have reported on the usefulness of temporal subtraction CT (TSCT) techniques for the detection of spinal bone metastases [5, 6]. TSCT depicted the areas where temporal changes existed as clear color differences compared to the previous CT, contributing to improved detection and evaluation efficiency. These previous studies have also reported problems with artifacts caused by misalignment during image registration; however, the use of HRCT with a slice thickness < 1 mm in the evaluation of the temporal bone enables the creation of remarkably clear TSCT images with few artifacts.

It is notable that there have been no reports on TSCT methods for temporal bone CT. Therefore, purpose of this study was to investigate the usefulness of TSCT of temporal bone CT for the detection of the recurrence/residual of middle ear cholesteatoma.

## Materials and methods

This retrospective study was approved by the institutional review board and ethics committee. Since this was a retrospective study, the requirement for informed consent was waived.

### Patient selection

**Fig. 1:**
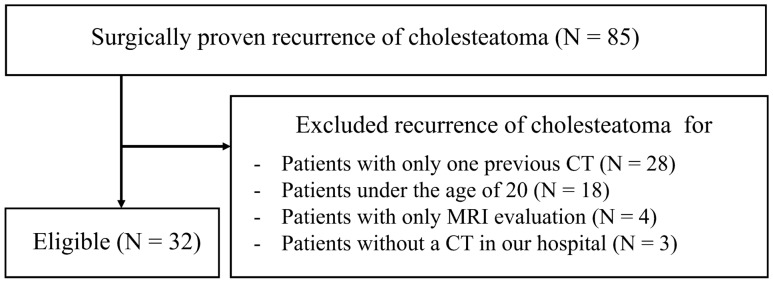
Flow diagram summarizing the patient selection

Consecutive patients who underwent surgery for recurrent/residual middle ear cholesteatoma between April 2016 and August 2020 were retrospectively evaluated. All patients had surgically proven recurrent/residual lesions. Patients with temporal bone HRCT for preoperative evaluation for recurrent/residual treatment and postoperative (primary lesion) CT performed more than 6 months ago with the same scan range were included. Patients younger than 20 years were excluded. Patients with only one CT performed between the primary surgery and the surgery for recurrent/residual lesions were excluded. Thirty-two patients matched the selection criteria (Fig. 1). Consecutive patients with previous surgery for primary middle ear cholesteatoma, with at least 2 years of follow-up between April 2016 and August 2018, with at least 2 postoperative CTs, and clinically negative for recurrent/residual were included as the recurrent/residual negative group.

### HRCT

HRCT of the temporal bone without contrast was performed using a 64-multidetector CT scanner (SOMATOM Perspective, Siemens AG, Munich, Germany). The CT scans in this study were performed in the supine position. The scan angle was set by referring to the orbitomeatal line. The scanning parameters were as follows: collimation, 64 × 0.6 mm; rotation time, 1.0 s; detector-row width, 0.6 mm; pitch, 1.0; and scanning field of view (FOV), 25 cm. The peak tube voltage was 130 kVp. The reconstruction parameters were as follows: section thickness, 0.6 mm and 0.6-mm reconstruction in the axial plane. The CT threshold was adjusted with the bone window setting (window center and window width were fixed at 700 and 4000). All HRCT imaging was performed with the same acquisition and reconstruction protocol. All HRCTs were without prominent artifacts, allowing for image registration and image evaluation as subsequently described.

### TSCT

TSCT imaging was generated by Fusion Viewer, a feature included in a 3-dimension (3D) image analysis system (SYNAPSE VINCENT; FUJIFILM Medical Co., Ltd., Tokyo, Japan) (https://synapse.fujifilm.eu/synapse-3d-apllications.html). Images were automatically registered in 3D space using normalized mutual information [7]. The practicality and robustness of this technique have been validated using anatomical MR brain images with added noise and distortion [7]. TSCT images were created by subtraction between the two images of the registration result. TSCT images were created based on the previously described HRCT axial images with an FOV of 25 cm including the bilateral temporal bone regions to prevent mis-registration as much as possible. Temporal decreases in CT values were visualized as negative values (black color) and increases as positive values (white color) in the TSCT images. All TSCT images were created in about one minute each.

### Image analysis

**Fig. 2:**
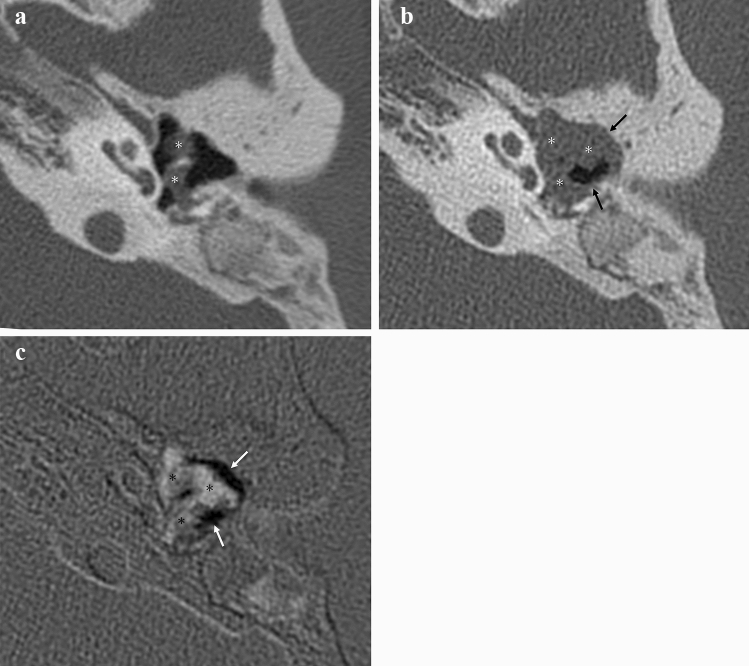
Postoperative recurrence of left pars flaccida cholesteatoma with relatively significant bone erosive changes. Postoperative temporal bone CT (**a**) about 9 years ago shows a soft tissue density (*) in the left tympanic and mastoid cavity. Temporal bone CT (**b**) before surgery for recurrence/residual shows increased soft tissue density (*) and adjacent progressive bony erosive changes (black arrows). In TSCT (**c**), progressive bone erosive changes are visualized as distinctive black areas (white arrows), and areas of increasing soft tissue density are visualized as white areas (*). TSCT, temporal subtraction CT

**Fig. 3:**
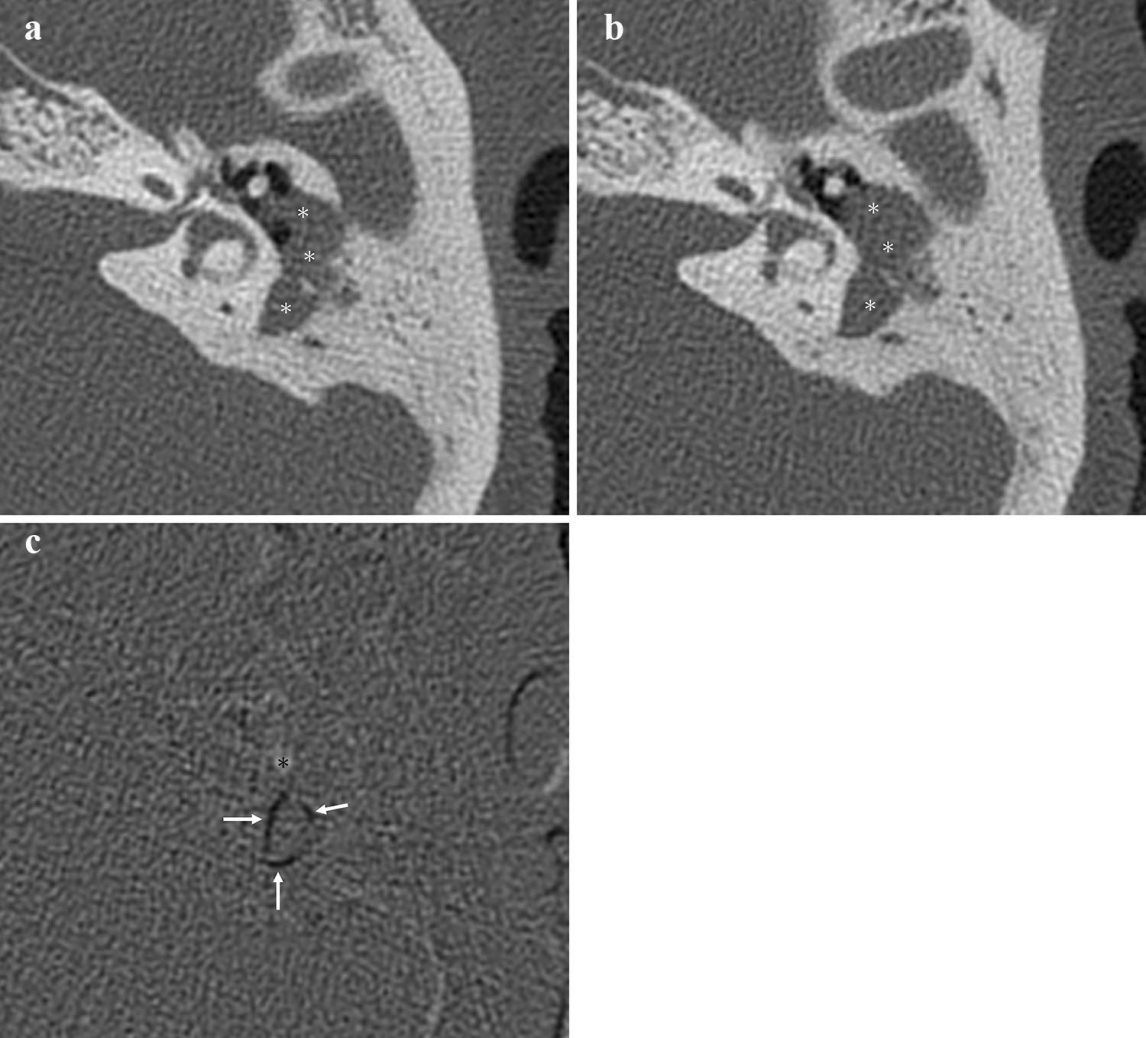
Postoperative residual lesion of left pars flaccida cholesteatoma with relatively slight bone erosive changes. Postoperative temporal bone CT (**a**) about 1 years ago shows a soft tissue density (*) in the left tympanic and mastoid cavity. Temporal bone CT (**b**) before surgery for recurrence/residual shows increased soft tissue density (*) and adjacent progressive bony erosive changes (black arrows). In TSCT (**c**), progressive bone erosive changes are visualized as distinctive black areas (white arrows), and areas of increasing soft tissue density are visualized as white areas (*). TSCT, temporal subtraction CT

Two serial CT scans from 32 patients with recurrent/residual middle ear cholesteatoma lesions and 14 patients in the recurrent/residual negative group were used for the image interpretation set, as previously described. Progressive bone erosive change on temporal bone CT is considered positive for recurrence/residual if there is progressive erosive change in bone structure adjacent to soft tissue density in tympanic cavity on the current CT compared to the previous CT (Figs. 2a, b, 3a, b). The TSCT is displayed alongside the synchronized and linked conventional temporal bone CT. The presence of progressive bone erosive changes is determined when TSCT shows prominent focal low density (compared to the low density seen due to slight misalignment in the temporal bone in the same slice) adjacent to areas of soft tissue density on the reference conventional CT (Figs. 2c, 3c). A decrease in the amount of soft tissue density indicating postoperative or other changes is also seen as a localized low density when assessed by TSCT alone, however, the reference conventional CT shows adjacent air concentrations, and such cases are not judged to be progressive bone erosive changes. Two radiologists (10 and 13 years of experience) were requested to evaluate these 46 patients for the presence of bone erosive changes. Two radiology residents (2 and 3 years of experience) also participated in the study. All CT images were displayed using the previously mentioned 3D image analysis system. Two reading sessions were held. The order of cases in each reading session was randomized. Readers were not informed of the patient's gender, age, or purpose of the examination. The first reading sessions were performed using the original pair of serial CT images without TSCT. The second reading session was performed at least 4 weeks after the first session. The second reading session was performed using the TSCT images plus the original CT images synchronized and linked. The reading time for each session was also recorded.

### Statistics

The detection rates between only two serial temporal bone CT scans and temporal bone CT and TSCT for the presence of bone erosive changes were compared by the McNemar test. The sensitivity and specificity of the finding of bone erosive change for detecting recurrent/residual middle ear cholesteatoma were calculated in the two serial temporal bone CT scans alone, or temporal bone CT + TSCT. The reading times by two serial temporal bone CT scans alone and that of the readers with temporal bone CT + TSCT were compared by the Mann–Whitney *U* test. A *p* value of less than 0.05 was considered to indicate statistical significance. All statistical analyses were performed using R version 3.6.1 (R Foundation for Statistical Computing, Vienna, Austria).

## Results

Thirty-two patients (21 males, 11 females; age range, 20–67 years; mean age [± SD], 43.1 ± 11.2 years) with recurrent/residual middle ear cholesteatoma and 14 patients (6 males, 8 females; age range, 35–68 years; mean age [± SD], 52.2 ± 8.6 years) in the recurrent/residual negative group were included in the study. In the recurrent/residual middle ear cholesteatoma group, the primary disease of the first surgery was pars flaccida cholesteatoma in 29 patients, pars tensa cholesteatoma in 2 patients, and unknown in 1 patient, and in the recurrent/residual negative group, the primary diseases were pars flaccida cholesteatoma in 12 patients, and pars tensa cholesteatoma in 2 patients. Postoperative cholesteatoma type was recurrent lesion in 21 patients, residual lesion in 9 patients, and residual and recurrent lesion in 2 patients. The interval time between CT scans ranged from 220 to 3434 days, with a median of 399 days, in the recurrent/residual middle ear cholesteatoma group and from 697 to 1287 days, with a median of 820 days, in recurrent/residual negative group. Evaluable TSCT images were successfully created in all cases. Patient characteristics are summarized in Table 1. The age and CT scan interval of recurrent/residual positive group were significantly lower than that of the negative group, and there was no significant difference in the male/female ratio (Table 1).

**Table 1:** Characteristics of the patients

	Recurrent/residual positive group	Negative group	Univariate *p *value
Sex			
Male	21	6	0.199^a^
Female	11	8	
Age	41 (20–67)	54 (35–68)	0.009^b^
CT scan interval day time	399 (220–3434)	820 (697–1287)	0.027^b^

Values are shown as median (minimum–maximum) in age and CT scan interval day time

^a^Fisher’s exact tests were used

^b^Mann–Whitney *U* tests were used

In all four readers, TSCT + CT significantly improved the detection of bone erosive changes over CT-only evaluation in recurrent/residual middle ear cholesteatoma (17.4–41.3% vs. 37.0–58.7%, *p* = 0.008–0.046) (Table 2). In all four readers' evaluation of recurrent/residual middle ear cholesteatoma cases, there was no significant difference in the CT scan interval between the positive and negative finding groups on TSCT + CT (*p* = 0.23–1.00). Sensitivity and specificity for the detection of recurrent/residual middle ear cholesteatoma judged by bone erosive changes and read time per case are summarized in Table 3. The mean sensitivity and specificity of TSCT + CT for experienced radiologists were 0.77 and 1.00, respectively, and 0.52 and 0.97 without TSCT, respectively. The mean sensitivity and specificity of TSCT + CT for residents were 0.64 and 1.00, respectively, and 0.41 and 1.00 without TSCT, respectively. Sensitivity showed an increase in all readers (Table 3). Among all readers, there were no false-positive cases of TSCT + CT; however, there were 5–15 false-negative cases (false-negative rate 0.16–0.47). The use of TSCT in all readers significantly reduced the reading time per case (*p* < 0.001–0.007) (Table 4).

**Table 2:** Number and detection rate of progressive bone erosive changes in readers

	CT without TSCT	CT with TSCT	Univariate *p *value^a^
Board-certificated radiologist 1	19/46 (41.3%)	27/46 (58.7%)	**0.027**
Board-certificated radiologist 2	15/46 (32.6%)	22/46 (47.8%)	**0.046**
Resident 1	8/46 (17.4%)	17/46 (37.0%)	**0.008**
Resident 2	18/46 (39.1%)	24/46 (52.2%)	**0.041**

Significant *p* values are indicated in bold (significance considered *p* < 0.05)

*TSCT* temporal subtraction CT

^a^McNemar’s test were used

**Table 3:** Sensitivity and specificity for detecting recurrent/residual middle ear cholesteatoma in readers

	CT without TSCT		CT with TSCT	
	Sensitivity	Specificity	Sensitivity	Specificity
Board-certificated radiologist 1	0.56	0.93	0.84	1.00
Board-certificated radiologist 2	0.47	1.00	0.69	1.00
Resident 1	0.25	1.00	0.53	1.00
Resident 2	0.56	1.00	0.75	1.00

*TSCT* temporal subtraction CT

**Table 4:** Reading time in readers (seconds)

	CT without TSCT	CT with TSCT	Univariate *p* value^*a*^
Board-certificated radiologist 1	52.5 (11–240)	27 (6–116)	** < 0.001**
Board-certificated radiologist 2	56.5 (21–100)	19.5 (7–60)	** < 0.001**
Resident 1	54.5 (16–110)	18 (9–86)	** < 0.001**
Resident 2	42 (9–167)	20 (8–120)	** < 0.001**

Values are shown as median (minimum–maximum). Significant *p* values are indicated in bold (significance considered *p* < 0.05)

*TSCT* temporal subtraction CT

^a^Mann–Whitney *U* tests were used

## Discussion

In the diagnosis of recurrence/residual of middle ear cholesteatoma after primary surgery, the combination of TSCT and conventional CT significantly increased the detection of bone erosive changes compared to that in conventional CT alone, and showed increased sensitivity for the detection of recurrence/residual lesions regardless of the experience of readers. The reading time per case was also found to be significantly reduced in all readers. This novel technique has shown the potential to improve the performance of radiologists in detecting postoperative recurrence/residual of middle ear cholesteatoma.

The incidence of postoperative recurrence or residual of middle ear cholesteatoma is relatively high (5-year recurrence rate of approximately 15%) [8]; therefore its diagnosis is clinically important. In the past, second-look surgery was used to evaluate the recurrence or residuals; however, as an alternative, diagnostic imaging has become the predominant method. The evaluation of recurrent/residual lesions by MRI and DWI is widely used as a reliable modality because of its relatively simple evaluation and high diagnostic accuracy. However, CT has a higher spatial-resolution, more detailed anatomical assessment ability and increased penetration compared to MRI, and is used frequently for postoperative follow-up for cholesteatoma. However, temporal bone HRCT increases the difficulty in understanding the complex anatomical structures for some readers with low reading experience. The results of this study showed that the combined use of TSCT was useful in contributing to the increased detection rate of progressive bone erosive changes indicative of recurrent/residual cholesteatoma. Furthermore, the sensitivity of detecting recurrence/residual lesions changes increased regardless of the experience of the reader. This indicates that the method is highly versatile and can be used regardless of the experience as a radiologist. MRI and non-EPI DWI has very high sensitivity and specificity of 89.8% and 94.6% for detecting postoperative recurrence/residual of cholesteatoma [2]. However, MRI has issues related to convenience and cost compared to CT; therefore, there is diagnostic value in using CT to improve detection rates in postoperative cholesteatoma. Although TSCT is not a substitute modality for DWI, it can be a great adjunct in facilities that do not have MRI, cannot perform DWI for cholesteatoma detection, or cannot perform MRI each time to evaluate for recurrence/residual. On CT, an increase in soft tissue density and progressive bone erosive changes compared to the previous CT indicates recurrence/residual of cholesteatoma. In particular, it is difficult to differentiate inflammatory changes from recurrence/residual when there is only an increase in soft tissue density, and therefore the finding of progressive bone erosive changes is the definitive CT finding of recurrence/residual. However, the findings of progressive bone erosive changes are often subtle and difficult to detect by serial CT comparison. The current TSCT evaluation was designed to more clearly and sensitively detect progressive bone erosive changes. These advantages may have influenced the detection of progressive bone erosive changes, increased sensitivity of detecting recurrence/residual lesions, and reduced reading time.

Previous studies have reported the benefit of temporal subtraction techniques on lung nodules [9, 10] and subtraction iodine imaging on head and neck cancer [11, 12]. There are also reports on the usefulness of the TSCT technique for the detection of spinal metastases, including improved lesion detection and reduced reading time [5, 6]. In this TSCT study, as in previous reports, an improvement in lesion detection and work time was observed. In addition, since TSCT can be created using normal routine CT, it does not require new radiation exposure or patient labor, which is considered to be a great benefit. For these reasons, TSCT might be an additional method to the ordinary interpretation in postoperative cholesteatoma.

Reading time is significant for radiologists because it affects both quality of life and the quality of the readings [13, 14]; the number of readings has increased with the recent widespread use of multi-slice CT and MRI [15]. In addition, radiologists are required to perform tasks other than reading, including communicating with clinicians [16, 17], and the shortage of radiologists in some countries [18] implies that reduction in reading time and improvement in the efficiency of reading are currently important. Temporal bone CT can also increase the reading time for some readers with low reading experience who do not understand the complex anatomy. In the current study, the temporal subtraction techniques significantly reduce the reading time for this task, further improving the efficiency of this task in detecting progressive bone erosive changes. This improvement is also independent of experience, which is noteworthy for its versatility and usefulness regardless of the experience.

The creation of TSCT images is one of the applications of a 3D image analysis system that is commercially available, and can be performed at facilities with installed equipment without the need to develop special algorithms. Furthermore, it can be created at the reading device of a radiologist as well as at our institution. The TSCT images created in this study are extremely easy to create. It should be noted that the position alignment of the past and present images is fully automated, and the images can be created in about 1 min, including the calculation of the difference. In temporal bone CT, a high-resolution image is essential for optimum visual assessment as the various anatomical structures are clustered in a small area [19, 20]. In this study, TSCT was designed to visualize the difference between two HRCT images with a thin slice of 0.6 mm (while performing a rigid registration on 3D space), resulting in the high diagnostic performance of TSCT. To date, there are no publications evaluating the subtraction method with the 3D image analysis system used in this study.

There were several limitations to our study. The number of patients included in our study was small, and it was a retrospective study design. There is a limitation that the criteria based on the evaluation of head and neck radiologists used as the gold standard of bone erosive changes are not necessarily an absolute standard, because it is not possible to determine whether progressive bone erosive changes were actually present on CT as a surgical or pathological finding. TSCT showed a false-negative rate of 0.16–0.47 in detecting recurrence of middle ear cholesteatoma, and negative findings on TSCT might not necessarily exclude the presence of recurrence. Even if the TSCT finding is positive, the possibility that it reflects bone erosive changes caused by postoperative cholesterol granuloma [21] cannot be excluded. Although there were no cases of surgically confirmed cholesterol granulomas alone during the period covered in this study, cases in which positive TSCT findings were detected due to cholesterol granulomas co-existing with cholesteatoma may have been included. To prevent mis-registration, TSCT was created based on the axial image of the HRCT, which had a relatively wide FOV including the bilateral temporal bone regions, and thus coronal TSCT images could not be evaluated. Patients under 20 years of age were not included in this study as the change in size of the entire temporal bone region due to growth could produce artifacts in some preliminary tests, and the development of temporal bone has significant regional differences in the ages of maturation [22]. Therefore, since congenital cholesteatoma is more common in younger patients, the TSCT method may not have significant efficacy in such cases. Because our final objective is to apply the TSCT method to patients with postoperative cholesteatoma at the initial evaluation, we have a selection bias in our study population. The number of cases in the recurrent/residual negative group (control group) is smaller than that in the recurrent/residual group. This is because patients without clinically suspected recurrent/residual cholesteatomas are less likely to have at least two serial CT studies at our institution. All readers first read without TSCT and then with TSCT. It is expected that the read order bias can be minimized by setting the read interval for the washout period. However, read order bias may not be completely eliminated.

## Conclusion

The TSCT obtained using serial CT improves the depiction of newly occurring progressive bone erosive changes, and detection sensitivity and reading time in postoperative recurrent/residual lesions of middle ear cholesteatoma.
